# The Use of Thermal Imaging in the Evaluation of Temperature Effects of Radiotherapy in Patients after Mastectomy—First Study

**DOI:** 10.3390/s21217068

**Published:** 2021-10-25

**Authors:** Agnieszka Baic, Dominika Plaza, Barbara Lange, Marta Reudelsdorf-Ullmann, Łukasz Michalecki, Agata Stanek, Krzysztof Ślosarek, Armand Cholewka

**Affiliations:** 1 Department of Medical Physics, A. Chełkowski Institute of Physics, University of Silesia, 75 Pułku Piechoty 1A St., 41-500 Chorzów, Poland; armand.cholewka@gmail.com; 2 Radiotherapy Planning Department, Maria Skłodowska—Curie National Research Institute of Oncology Gliwice Branch, Wybrzeze Armii Krajowej Street 15, 44-102 Gliwice, Poland; dominikaplaza1@gmail.com (D.P.); barbara.lange@io.gliwice.pl (B.L.); marta.reudelsdorf@io.gliwice.pl (M.R.-U.); krzysztof.slosarek@io.gliwice.pl (K.Ś.); 3 Department of Radiation Oncology, University Clinical Center of the Medical University of Silesia, Medyków 14 St., 40-572 Katowice, Poland; lmichalecki@uck.katowice.pl; 4 Department and Clinic of Internal Medicine, Angiology and Physical Medicine, Faculty of Medical Sciences in Zabrze, Medical University of Silesia, Batorego 15 St., 41-902 Bytom, Poland; astanek@tlen.pl

**Keywords:** radiation therapy, thermography, breast cancer

## Abstract

The aim of the study was to evaluate the temperature parameter of the breast area in patients undergoing radiotherapy at various intervals. The relationship between temperature changes on the patient’s skin and the time after the end of radiotherapy was studied. Measurements with a thermal imaging camera were performed in a group of twelve volunteers. Six of them were healthy women who did not have thermal asymmetry between the breasts, whereas six were diagnosed with breast cancer and underwent mastectomy due to the advanced stage of the disease. The patients were qualified for radiation therapy. Thermographic examinations were performed before treatment, two months later and then six months after the end of the treatment. Temperature differences between the healthy breasts and the treated areas were assessed. Additionally, the correlation between a patient’s skin temperature changes and the time after the end of radiotherapy was analyzed. The highest skin temperature increase (1.47 °C) was observed 6 months after the end of RT compared to the measurement before treatment. It seems that thermovision may bring a new tool for quantitative analyses of the temperature effects of radiotherapy.

## 1. Introduction

The leading cause of cancer death in female patients is breast cancer. The most common risk factors include: age, area of residence, reproductive factors, menopausal age, family history, occurrence of benign breast changes and lifestyle. The symptoms of breast cancer depend on the stage of its development: in its early stages, it is often asymptomatic, while the advanced stage depends on the extent of the local lesions and the location of the metastatic foci. The most common treatments of breast cancer include combination therapy treatments: local methods (surgery and radiotherapy) and systemic treatments (chemotherapy, hormone therapy and biological methods). The choice of treatment method depends on many factors—prognostic and predictive ones. Radiotherapy is based on the use of high-energy ionizing radiation aimed at destroying cancer cells with as little damage to healthy cells as possible. Two main types of radiotherapy are used in the treatment of breast cancer: teleradiotherapy (external radiation) and brachytherapy (direct radiation to cancer cells). Teleradiotherapy is used in the form of several minute-long cycles (the irradiation time depends on the treatment plan performed) for a period of 3–5 weeks.

Indications to use radiation therapy after mastectomy are metastases to four or more axillary lymph nodes, size T3 and T4 primary tumors, nonradical surgery and metastases to one to three axillary lymph nodes if they are accompanied by additional risk factors for locoregional recurrence. Mostly, irradiation covers the area of the chest wall after the removal of the breast and the area of supraclavicular, axillary and parasternal nodes. Irradiation after mastectomy uses photon–electron techniques or IMRT photon techniques (static or dynamic (V-MAT)). The irradiation area is set on the basis of computed tomography images. Usually, patients are treated with a total dose of 50 Gy in 25 fractions of 2 Gy or 45 Gy in 20 fractions of 2.25 Gy each.

The use of teleradiotherapy carries the risk of adverse effects on the skin and subcutaneous tissue, manifested as radiation dermatitis. Then, the skin reaction appears as erythema. This reaction may also take the form of: peeling, swelling, exceptional skin necrosis or ulceration (depending on the duration of radiotherapy and the dose received), as well as fibrosis. There are also systemic symptoms: malaise, weakness, drowsiness, loss of appetite, nausea, vomiting and diarrhea. Depending on the time of the appearance of the skin changes, we can distinguish early and late reactions. An early reaction appears several weeks after the start of the treatment, while late reactions usually appear several months after the end of irradiation. Both early and late reactions significantly deteriorate the patient’s quality of life. An important stage of the procedure, which determines the implementation of proper care and treatment, is the assessment of the severity of the changes in the skin. In 2003, the National Cancer Institute developed the Common Terminology Criteria for Adverse Events (NCI CTCAE)—a five-point scale to monitor and evaluate the local radiation reaction. The negative effects caused by radiation therapy may persist from several weeks to even several years after the end of the treatment [1,2,3,4,5,6,7,8,9,10,11,12,13,14,15,16,17].

Thermography is one of the medical imaging techniques that combines both morphological and functional imaging features. The temperature distribution on the surface of the human body depends on the temperature of the internal organs, the thermal conductivity of muscle and adipose tissues and the emissivity of the skin. Disturbances in heat production and dissipation caused by diseases of a specific organ can be easily captured on a thermovision image of patients. The thermal image of an affected organ will differ significantly from that of a healthy organ. It should be noted that thermographic examinations of the human body are completely noninvasive and do not cause any harm to the patient’s body. Due to that, these are used as a diagnostic method in many fields of medicine, including oncology, in relation to skin, bone and mammary cancer [18,19,20,21,22,23,24,25,26,27,28,29,30,31,32,33,34,35,36,37,38,39,40,41,42,43,44,45].

This paper aims to present the use of thermal imaging to describe the thermal response of breast tissue to radiation therapy and to propose a new method that may be useful to control the risk of developing radiation dermatitis. Previous studies have described the process of skin toxicity arising after radiotherapy, which, in most cases, manifests by the formation of erythema on the skin but does not show the changes that occur in individual months after radiotherapy and how long they persist on the skin. These changes are determined by the increased temperature of the diseased breast in relation to the healthy breast. The aim of the study is controlling the patient for a few months after radiotherapy in order to increase the awareness of the mechanisms of radiation actions on tissues and, also, what is more important, the improvement of the patient’s comfort after radiotherapy [46,47]. Two hypotheses were adopted in the presented work. The H0 hypothesis sates that the patient’s breast/chest temperature does not change after radiotherapy in patients after mastectomy. In this case, the patient’s thermal images will not vary with time. An alternative H1 hypothesis states that the temperatures of patients after mastectomy change after treatment with radiotherapy. Under these assumptions, a patient’s thermal images will change over time. Rejecting the H0 hypothesis and accepting an alternative hypothesis will confirm the usefulness of thermal imaging in assessing temperature changes caused by ionizing radiation.

## 2. Materials and Methods

The preliminary research included 6 patients after mastectomies treated with radiotherapy from 2 to 6 months after treatment and 6 healthy women. Patients participating in the study were in groups I and II of the NCI CTCAE scale aimed at assessing the severity of changes in the skin.

The thermal imaging was carried out in a specially prepared room in which the temperature was kept at 22 ± 1 °C, while the humidity was between 40% and 45%. The study used a specialized FLIR Systems (Teledyne FLIR LLC, Wilsonville, OR, USA) E60 thermal imaging camera with a detector resolution of 320 × 240 pixels, the thermal sensitivity of which was 0.05 K. The thermographic camera used in the work was calibrated and validated on the basis of the international ISO norms and standards, which are related to the technical aspects of thermography, such as the specification of used measuring devices and systems, calibration of thermal imaging equipment and the minimum requirements for measuring devices [48,49,50].

Before the study, the patients received a qualifying questionnaire for the study and appropriate consents and were informed about the methods of the experiment. An interview was conducted with the patients to assess the influence of additional factors, such as age, genetic predisposition, previous procedures and pregnancy. During the preparation for the study, the patients were left naked from the waist up for 20 min, which was referred to as the acclimatization process to the temperature of the measuring room. In accordance with the guidelines, each patient had a thermal image taken with raised hands of three projections—front, left side and right side [25,26,27,28,29,30]. Only the front projections were taken into account for the further analyses.

In order to analyze the thermograms, we used ThermaCam Researcher Pro 2.10 (Teledyne FLIR LLC, Wilsonville, OR, USA). To interpret the results more broadly, a statistical analysis was performed in Statistica 10, where the confidence interval was 0.95. All the parameters that were obtained were tested for normality firstly. To determine the significant statistical value of the research, the Student’s *t*-test was performed. The results were presented using graph boxes. Additionally, to analyze the temperature changes over time on the body surface, the correlation between the temperature values and time after radiotherapy was done using Pearson’s coefficient.

The aim of the study was to assess the temperature parameters of the breast area in patients who underwent radiotherapy at various intervals (from 2 to 6 months after treatment). Additionally, the relationship between the temperature changes on the patient’s skin and the time after the end of radiotherapy were checked. All patients were treated by radiotherapy due to breast cancer.

The study project was approved by the Bioethics Committee at the Oncology Center—Maria Skłodowska-Curie Institute in Warsaw on 6 October 2016, as confirmed by opinion no. 38/2016.

**Figure 1:**
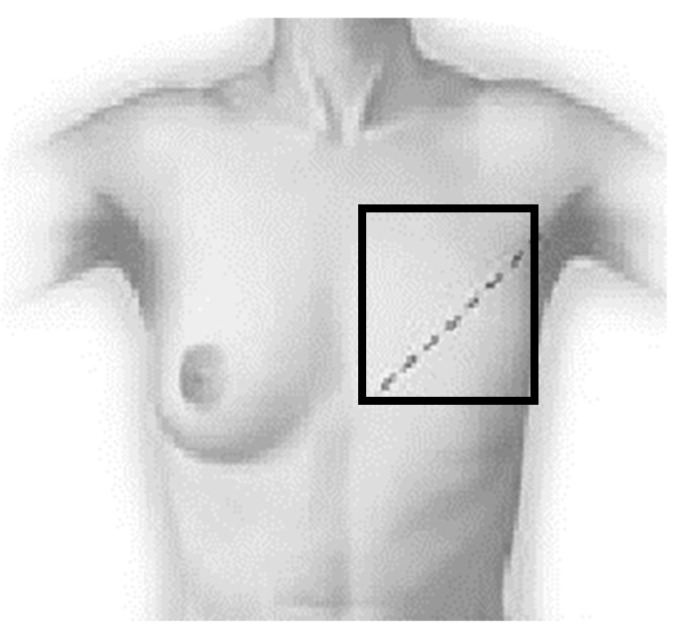
The scheme of the breast area drawings in all the thermograms.

In the case of a mastectomy, the entire irradiated area was assessed, i.e., the area of the chest wall and the lymph nodes (the area marked with a square (Figure 1) was used for the thermal analysis). This square was of the same size and in the same place in every thermograms, which allowed us to accurately determine the temperature changes over time.

## 3. Results

**Figure 2:**
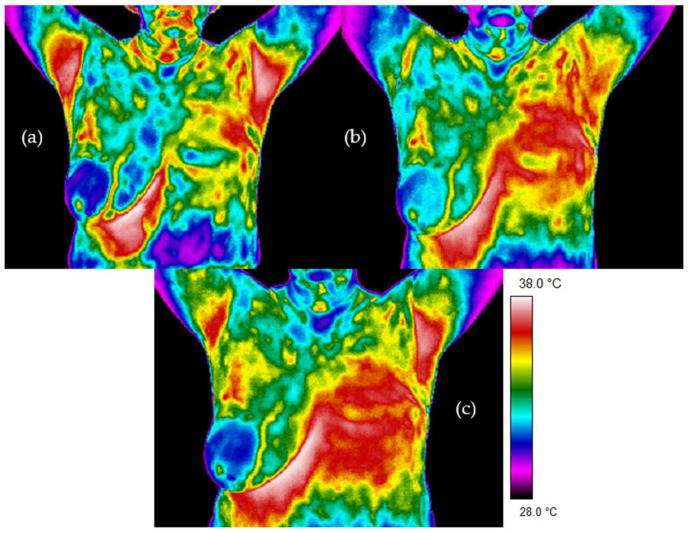
Examples of thermograms for patient 1: (**a**) before radiotherapy, (**b**) 2 months after radiotherapy and (**c**) 6 months after radiotherapy.

**Figure 3:**
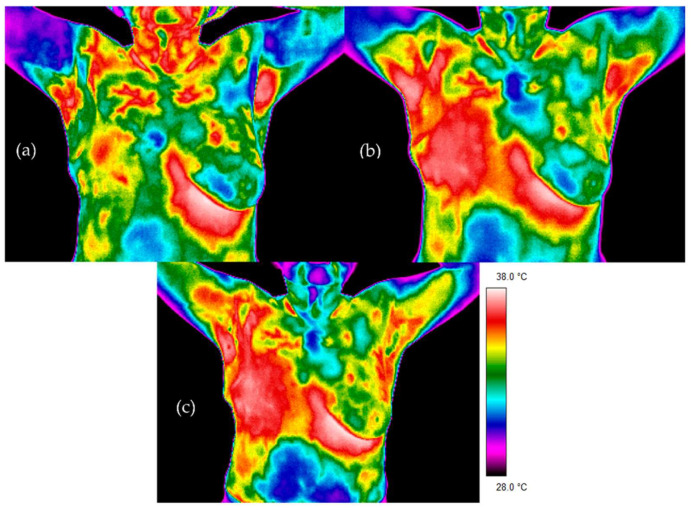
Examples of thermograms for patient 2: (**a**) before radiotherapy, (**b**) 2 months after radiotherapy and (**c**) 6 months after radiotherapy.

**Figure 4:**
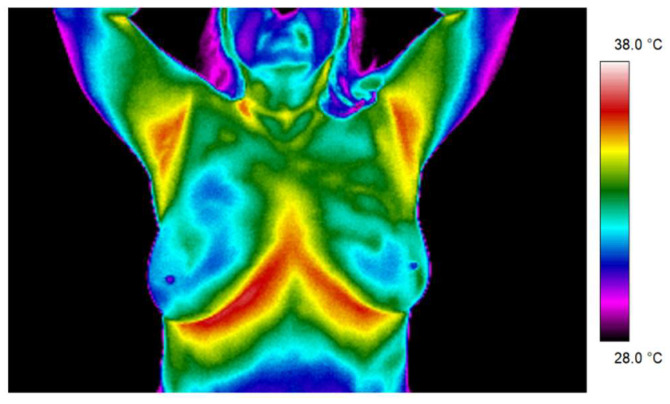
The exemplary thermogram for healthy women.

Figure 2 and Figure 3 present thermal images for representative patients who finished radiotherapy in a different time. The temperature range was from 28 to 38 °C for each patient. Patient number 1 took radiotherapy on the left breast mastectomy area. Two months after the treatment, thermal asymmetry could clearly be seen. Moreover, the temperature increased after additional time following RT, which is also visible in the thermograms. In the case of patient number 2, the right side was irradiated. We can see a temperature rise that was greater 6 months after the end of the treatment compared to the thermograms taken after two months following RT. One can clearly see the differences in the heat maps between the irradiated breast and healthy breast (nonirradiated one). The mean temperature value in the defined area, after breast mastectomy and before radiotherapy, was 34.37 °C. Two months after the end of radiotherapy, the temperature was raised by 0.83 °C. It should be noted that a similar effect was observed in each of the studied patients. Additionally, after 6 months after the treatment, the temperature of the treated area increased by as much as 1.47 °C compared to the first measurement. The highest temperature of the irradiated breast area could be observed in patients who finished radiotherapy 6 months before. On the other hand, it is noteworthy that, for healthy women, the average temperature difference obtained between the breasts was 0.20 °C and the representative thermal image for healthy women from the control group is presented in Figure 4.

The figure below (Figure 4) presented the exemplary thermogram for healthy women.

For deeper insights into the problem, the temperature data was collected in tables and analyzed in detail.

The table below (Table 1) presents the results for six post-mastectomy patients treated with radiotherapy for whom thermal imaging camera measurements were performed before radiotherapy and 2 and 6 months after the end of treatment. The table also shows the results for six healthy women. It can be seen that the highest temperature increase was observed 6 months after the end of RT compared to the measurements before treatment.

A deeper analysis showed that the temperature difference was the highest for 6 months after RT, as shown in Table 2.

All the parameters that were obtained were tested for normality firstly. In order to determine the significant statistical value of the research, the Student’s *t*-test was performed.

**Figure 5:**
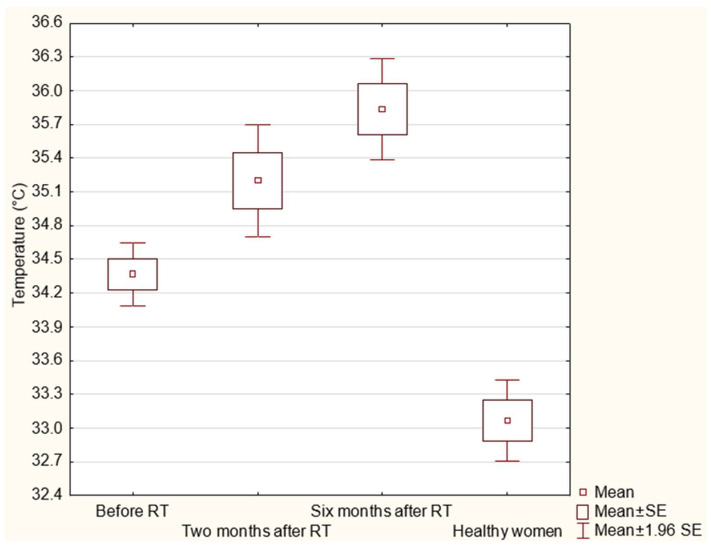
Breast area temperature values before radiotherapy, two months after treatment and six months after treatment at the same area for all patients and the healthy control group.

The temperature differences for the same breast area before radiotherapy and 2 and 6 months after treatment were statistically significant at *p* < 0.05, as shown in the graph boxes in Figure 5. What is more, another analysis carried out in the study was the comparison of the breast skin temperature of patients before radiotherapy and six months after treatment with a control group of healthy women. Statistical significance (*p* < 0.05) was confirmed, as also shown in Figure 5.

## 4. Discussion

For many years, thermographic examinations have been used in various fields of medicine—for the diagnosis of diseases of temporomandibular joints, diseases of the musculoskeletal system, assessment of the extent of burns, skin diseases or rheumatology. The greatest advantage of thermography is its noninvasiveness, which allows us to repeat studies. What is more, it is harmless to humans and animals, with no risk of side effects. The best-known medical use of thermography is in breast cancer diagnostics in women. Inspired by that research, we decided to check whether thermography can be used in breast cancer to assess the effects of radiotherapy treatments. For a healthy person, cyclical daily temperature fluctuations are typical. The temperature depends on many factors. These include gender, age, mode of work, medications taken and other activities performed by the patient. It is therefore important to observe all the necessary steps and procedures that may cause an incorrect result when taking your body temperature. Radiation therapy destroys cancer cells with ionizing radiation.

The side effects caused by radiation therapy are radiation reactions. The risk of a reaction is increased in the case of combination therapy—the combination of correlation radiotherapy and chemotherapy. The radiation reaction is caused by the irradiation of healthy tissues in the treatment area. Unfortunately, it is not possible to undergo radiotherapy only on cancerous tissues. Ionizing radiation also affects unchanged cells. However, in order for the dose received by the patient to be safe for him and healthy cells to have time to rebuild, fractionation is used. The dose is administered five times a week at an interval of two days. Such a scheme developed over the years seems to be the most favorable. Healthy cells have the opportunity to regenerate damage during this time. Cancer cells that divide rapidly are more susceptible to ionizing radiation—they are said to be radiosensitive. Based on the available literature on the effects of radiotherapy on the body, as well as on the knowledge of the effects of radiation over time, it has been assumed that radiotherapy can cause changes in the body temperature that we can observe with thermovision [50,51,52,53].

**Figure 6:**
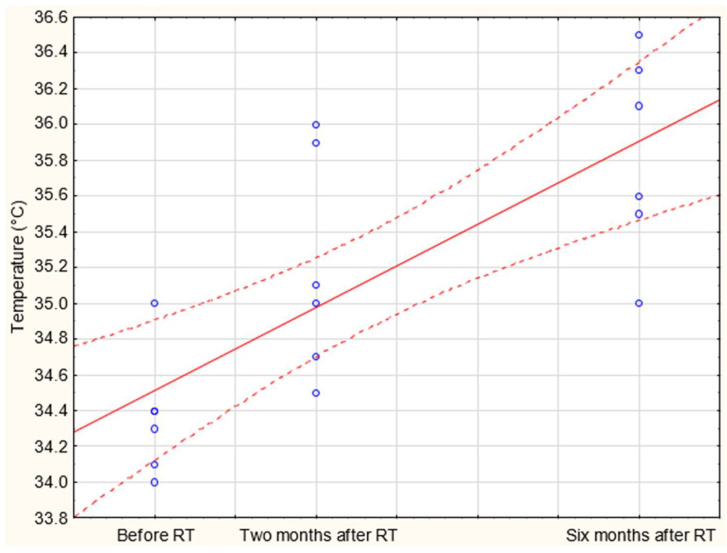
Correlation graph between the temperature values and time after radiotherapy.

In order to analyze changes in the temperature over time on the body surface, correlations between temperature values and time after radiotherapy were performed for the research groups. Pearson’s correlation coefficient was 0.77, and moreover, the relationships were statistically significant with *p* < 0.05. The coefficient of determination was 59%, respectively. It follows that 59% of the body surface temperature changes may explain or may be related to the parameter that is the time after radiotherapy. Based on the correlations shown in Figure 6, a positive relationship between the parameters could be seen, which showed that the temperature values increased with the length of time after radiotherapy. It was assumed that research with the use of thermovision confirmed the increase in temperature in the studied area after radiotherapy. This might reflect an increased temperature of the body observed in thermal images. Thermal imaging allows to assess temperature changes resulting from the influence of ionizing radiation on the body. In addition, it was examined how temperature shifts depend on the time after the end of treatment. During post-treatment observations, doctors will be able to evaluate the condition of the skin after radiotherapy, which will allow them to supply additional information, such as the patient’s sensitivity to radiation, the appearance of a radiation reaction and the size of the area where the temperature is altered. The temperature of skin is related to the blood supply, intensity of metabolism and edema. These factors result in metabolism changes, which are observed as changes in the skin’s temperature. It is obvious that the first skin reaction to irradiation will appear as a burn, which will be seen as a higher temperature than the areas not irradiated due to energy administered to the tissue. However, skin cells in treated areas will regenerate with time, so the metabolism should change, and thermal maps obtained in different time periods will differ. The performed studies gave us the possibility to see a significant increase of the treated area temperature 6 months after treatment, which may be connected to radiation dermatitis. An interesting finding is that, after six months, the temperature was higher than two months after treatment.

The pilot study, which was carried out on a small group of patients, suggested possibilities for using thermal imaging as a fast and safe tool for the evaluation of radiotherapy effects, and thermal imaging brings valuable confirmation of the metabolic processes taking place in radiated tissue, while the low power of the test due to the sample size did not allow us to reject the H0 hypothesis. In order to confirm the conclusions presented in the article, it is necessary to enlarge the research group.

## 5. Conclusions

The performed preliminary studies showed that thermovision gives us a possibility to evaluate the increase in temperature resulting from the body’s response to a radiation dose. The increase of temperature of the irradiated body area with time after radiotherapy has been obtained, and the increase correlates with a radiation reaction on the CTCAE scale. As thermal imaging is a noninvasive method, measurements can be repeated many times without exposing the patient. This allows checking the regeneration process of the skin after radiotherapy at different times. Such measurements may provide us with additional information about how long the regenerative process of tissues subjected to ionizing radiation lasts. The radiation reaction visible to the naked eye can be determined not only visually but by analyzing the thermograms, which seems to be a more accurate form of assessment.

Further studies are needed to confirm the usefulness of this method in controlling the temperature effects of radiotherapy treatments and its effects on irradiated skin months or even years after the treatments.

## Figures and Tables

**Table 1:** Results of breast temperatures for all patients and for the healthy control group.

Patients	Before RT (°C)	Two Months after RT (°C)	Six Months after RT (°C)	Healthy Women (°C)
1	34.00	34.50	36.30	32.50
2	34.40	35.10	35.50	33.20
3	34.10	34.70	35.00	33.70
4	35.00	36.00	36.50	32.60
5	34.40	35.90	36.10	33.30
6	34.30	35.00	35.60	33.10

**Table 2:** Temperature differences between the patients before RT and 2 and 6 months after RT.

Before RT and 2 Months after RT (°C)	Before RT and 6 Months after RT (°C)
0.83	1.47

## Data Availability

Not applicable.
